# Incentives of Using the Hydrodynamic Invariant and Sedimentation Parameter for the Study of Naturally- and Synthetically-Based Macromolecules in Solution

**DOI:** 10.3390/polym12020277

**Published:** 2020-01-31

**Authors:** Mandy Grube, Gizem Cinar, Ulrich S. Schubert, Ivo Nischang

**Affiliations:** 1 Laboratory of Organic and Macromolecular Chemistry (IOMC), Friedrich Schiller University Jena, Humboldtstraße 10, 07743 Jena, Germany; mandy.grube@uni-jena.de (M.G.); gizem.cinar@uni-jena.de (G.C.); ulrich.schubert@uni-jena.de (U.S.S.); 2 Jena Center for Soft Matter (JCSM), Friedrich Schiller University Jena, Philosophenweg 7, 07743 Jena, Germany

**Keywords:** conformation, (intrinsic) diffusion coefficient, (intrinsic) viscosity, (intrinsic) sedimentation coefficient, hydrodynamic invariant, sedimentation parameter

## Abstract

The interrelation of experimental rotational and translational hydrodynamic friction data as a basis for the study of macromolecules in solution represents a useful attempt for the verification of hydrodynamic information. Such interrelation originates from the basic development of colloid and macromolecular science and has proven to be a powerful tool for the study of naturally- and synthetically-based, i.e., artificial, macromolecules. In this tutorial review, we introduce this very basic concept with a brief historical background, the governing physical principles, and guidelines for anyone making use of it. This is because very often data to determine such an interrelation are available and it only takes a set of simple equations for it to be established. We exemplify this with data collected over recent years, focused primarily on water-based macromolecular systems and with relevance for pharmaceutical applications. We conclude with future incentives and opportunities for verifying an advanced design and tailored properties of natural/synthetic macromolecular materials in a dispersed or dissolved manner, i.e., in solution. Particular importance for the here outlined concept emanates from the situation that the classical scaling relationships of Kuhn–Mark–Houwink–Sakurada, most frequently applied in macromolecular science, are fulfilled, once the hydrodynamic invariant and/or sedimentation parameter are established. However, the hydrodynamic invariant and sedimentation parameter concept do not require a series of molar masses for their establishment and can help in the verification of a sound estimation of molar mass values of macromolecules.

## 1. Introduction

The rise of the macromolecular hypothesis [1] and, therefore, the conceptual existence of macromolecules can be traced back to Hermann Staudinger, who can be associated with the very initial studies of macromolecules at the beginning of the last century [2,3,4]. Staudinger may, as well, be associated with the very initial attempts at conceptualizing the intrinsic viscosity, the prime example of rotational friction phenomena in macromolecular hydrodynamics, the so-called Staudinger index, better known as the intrinsic viscosity [5]. In very simple words, the intrinsic viscosity may be described as the (isolated) macromolecular object’s contribution to the viscosity of a solution and is the prime example of rotational friction phenomena in solutions of macromolecules and colloids. 

As well, at the beginning of the last century, The Svedberg pioneered a technique, nowadays widely known as analytical ultracentrifugation [6,7,8]. This technique allows for the study of translational friction phenomena, known as sedimentation and diffusion. One of his doctoral students, Ole Lamm, developed a partial differential equation for coupled mass transport processes based on sedimentation and diffusion in sector-shaped cell volumes [9], typically utilized in the very early, as well as modern, analytical ultracentrifugation equipment. The measurement of sedimentation coefficients by sedimentation velocity experiments making use of analytical ultracentrifugation as well as diffusion coefficients decoupled from sedimentation, found their inception at this period of time as well [10]. 

With these selected major pioneering contributions to the field of research of macromolecular hydrodynamics, particularly important for this review, began the study of basic hydrodynamic characteristics of macromolecules and colloids in solution, the intrinsic viscosity, the (intrinsic) diffusion coefficient, and the (intrinsic) sedimentation coefficient. 

The perhaps most classical and contemporary power of macromolecular hydrodynamics is associated to the absolute determination of molar masses of synthetically- and naturally-based macromolecules, respectively their assemblies or aggregates in solution. Though often not admitted by analysts, depending on their field of research, this is anything else than a trivial task. In the cumbersome challenge of utilizing macromolecular hydrodynamics, that rely on basic physical principles in solution, one typically cannot rely on a single experiment. Macromolecular hydrodynamics may be considered a counterpart to the well-known (static) light scattering experiments for direct molar mass estimations [11]. In any case, and also due to the multiplicity of experiments necessary to determine absolute molar masses, the estimated results from any technique or combination of techniques are statistical in nature, particularly for disperse macromolecule populations [12,13]. Furthermore, the accuracy of molar mass estimations needs to be confirmed by a suitable orthogonal correlation of individually determined experimental parameters establishing their absolute value [12,13,14]. 

The modern field of macromolecular hydrodynamics is characterized by the existence of sophisticated high precision instrumentation that enables to study all parameters important for hydrodynamic characterization with high accuracy. Particularly, this concerns modern equipment, such as viscometers, densimeters, and analytical ultracentrifuges, allowing for efficient temperature control and sophisticated multi-detection concepts. Additionally, next to naturally occurring macromolecules, the modern field of synthetic macromolecular chemistry has come up with a multitude of macromolecular structures and chemistries in solution that, after synthetic tailoring, require the establishment of quantitative structure-property relationships. Last but not least, this also concerns the absolute molar mass, inaccessible by calibrated standard procedures, such as size-exclusion chromatography. 

It is exactly this advancement in synthetic concepts and opportunities that requires a retro-synthetic analysis of macromolecular structures in solution, reconciled by their fundamental molecular hydrodynamic properties. This is useful, as well, to determine absolute molar masses, reference-free and without instrument calibration based on standards. 

Typically, scientists in the interdisciplinary area concerning synthetic chemistry and advanced applications, seek to obtain an experimental understanding of their created macromolecular objects in solution, may these be molecularly dissolved or dispersed/aggregated. Flexible and tailored experimental settings are pivotal for such issues. Particularly, such situations could excellently be addressed by the utilization of scientific concepts of macromolecular hydrodynamics developed in the last century, which often remained unrecognized by the wider scientific community [1,15,16,17]. 

In the present short review article, we would like to recall the utilization of the very principles known from nature: diffusion, sedimentation, and the contribution of macromolecular or colloidal objects to the viscosity of solutions. The determined parameters, accessible by experimental methods of rotational and translational friction are briefly introduced, their seminal interrelation discussed, and the practical utility for deriving conclusions on diverse macromolecular structures is outlined. The fundamental interrelation emanates from experimental approaches from the area of macromolecular hydrodynamics and provide highly orthogonal insight by the establishment of the hydrodynamic invariant [17] and/or the sedimentation parameter [18,19]. We further evidence that, as judged from the comprehended literature data, the concepts can be applied for synthetically- and naturally-based macromolecules, as well as for synthetic variants thereof. Their establishment is based on the very prime hydrodynamic characteristics, i.e., (intrinsic) viscosity/concentration sedimentation coefficient, (intrinsic) sedimentation coefficient, and (intrinsic) diffusion coefficient of a particular macromolecular sample.

## 2. Parametric Considerations

A historically useful model concept in macromolecular physics is the mean square end-to-end distance of macromolecular chains, $\langle h^{2}\rangle$, more precisely of a freely jointed chain [1]. This can be considered as a statistical macromolecular descriptor determining hydrodynamic parameters of interest, i.e., the intrinsic viscosity, $\left[\eta \right]$, the intrinsic sedimentation coefficient, $\left[s\right]$, and the intrinsic diffusion coefficient, $\left[D\right]$, according to the following relations:

(1) $$\left[\eta \right]=\phi \frac{\langle h^{2}\rangle^{3/2}}{M}\;$$

(2) $$\left[s\right]=\frac{M}{N_{A}P\langle h^{2}\rangle^{1/2}}\;$$

(3) $$\left[D\right]=\frac{k_{B}}{P\langle h^{2}\rangle^{1/2}}$$

where M denotes the molar mass of the macromolecule, ϕ (in units ${\mathrm{mol}}^{-1}$) and P (dimensionless) are Flory hydrodynamic parameters, $N_{A}$ is the Avogadro constant, and $k_{B}$ is the Boltzmann constant.

In hydrodynamic experiments one typically seeks to determine these estimates, i.e., $\left[\eta \right]$ (Equation (1)), $\left[s\right]$ (Equation (2)), and $\left[D\right]$ (Equation (3)). Classical experiments to determine intrinsic viscosities, $\left[\eta \right]$ (Equation (1)), are those of solution viscometry at suitable dilutions of macromolecule solutions and extrapolation procedures to infinite dilution by, e.g., the Huggins and/or Kraemer relations, which yield intrinsic viscosities $\left[\eta \right]$ in units ${\mathrm{cm}}^{3}g^{-1}$ [20,21]. $\left[\eta \right]$ is representative of the hydrodynamic volume of the macromolecules (Equation (1)). Under particular conditions, $\left[\eta \right]$ (Equation (1)) can experimentally be assessed by: [2,20,21]

(4) $$\left[\eta \right]\equiv \underset{c\to 0}{lim}\frac{\eta_{r}-1}{c}\equiv \underset{c\to 0}{lim}\frac{ln\eta_{r}}{c}$$

where $\eta_{r}$ is the relative viscosity defined by the ratio of the viscosity of a solution at a certain macromolecule mass concentration, $\eta_{c}$, and the solvent viscosity, $\eta_{0}$, i.e., $\eta_{r}=\eta_{c}/\eta_{0}$. 

Classical experiments to determine sedimentation coefficients are those of sedimentation velocity measurements utilizing an analytical ultracentrifuge. The sedimentation coefficient, s, by experiment is defined by:

(5) $$s\equiv \frac{dx/dt}{\omega^{2}r}\;$$

where dx/dt is the radial displacement of the sedimentation boundary per unit time, ω is the angular velocity of the rotor, and r is the radial distance from the center of rotation. The sedimentation coefficient, s is typically reported in units Svedberg, S, equaling $10^{-13}\;s$ as follows from Equation (5). Modern software allows for the numerical solution of the Lamm equation on sedimentation velocity data. This opens the gate to the determination of sedimentation coefficients, s, and the diffusion coefficient, D, at a particular macromolecule solution concentration, c: [9,22]

(6) $$\frac{dc}{dt}=\frac{1}{r}\frac{\partial }{\partial r}\left[\left(D\frac{\partial c}{\partial r}-\omega^{2}rsc\right)r\right]$$

The determination of the intrinsic sedimentation coefficients, $\left[s\right]$, typically has to be performed via suitable extrapolation procedures to infinite dilution by first measuring the sedimentation coefficient, s, at several concentrations and by extrapolating to infinite dilution to determine $s_{0}$ according to the following relation:

(7) $$s^{-1}=\;s_{0}^{-1}\;\left(1+k_{s}c\right)\;$$

where $k_{s}$ is the concentration-sedimentation, i.e., (Gralen) coefficient [23]. $s_{0}$ subsequently allows the calculation of the intrinsic sedimentation coefficient $\left[s\right]$:

(8) $$\left[s\right]=\frac{s_{0}\eta_{0}}{\left(1-\upsilon \varrho_{0}\right)}\;$$

where $\left(1-\upsilon \varrho_{0}\right)$ is the dimensionless buoyancy factor with υ being the partial specific volume of the macromolecule in units ${\mathrm{cm}}^{3}g^{-1}$, and $\varrho_{0}$ the solvent density in ${\mathrm{gcm}}^{-3}$.

A peculiar opportunity of such extrapolation procedures and the case of typically existent non-ideality over suitable concentration ranges is, that $k_{s}$ in Equation (7) can also be expressed via the chain end-to-end distance, $\langle h^{2}\rangle$, of the macromolecules:

(9) $$k_{s}=B\frac{{\langle h^{2}\rangle }^{3/2}}{M}$$

where B (in units ${\mathrm{mol}}^{-1}$) is a parameter for the given macromolecule system. The experimental $k_{s}$ values in units ${\mathrm{cm}}^{3}g^{-1}$ (Equation (7)) then are the translational friction analogue for $\left[\eta \right]$ (Equation (4)), by noting the similarity between Equations (1) and (9).

The intrinsic diffusion coefficient, $\left[D\right]$, first requires the determination of the diffusion coefficient at infinite dilution, $D_{0}$, by measuring diffusion coefficients, D, at different dilutions and again extrapolating to infinite dilution according to the following relation:

(10) $$D=D_{0}\left(1+k_{D}c\right)$$

where $k_{D}$ is the concentration-diffusion coefficient. $D_{0}$ allows the calculation of the intrinsic diffusion coefficient, $\left[D\right]$:

(11) $$\left[D\right]=\frac{D_{0}\eta_{0}}{T}$$

where T is the absolute temperature in units K.

Summarizing from the set of Equations (1)–(11), particularly Equations (4), (7), (8), (10), and (11), these can be utilized for their interrelation, since all equations relate to the same hydrodynamic characteristics, i.e., hydrodynamic volume/size (Equations (1)–(3)) and/or the molar mass (Equations (1) and (2)). Though expressed for linear macromolecular chains, the validity of the experimental measurements can be checked for by the very same set of experimental approaches (vide infra). As well, real macromolecule systems are always non-ideal, i.e., the effects of hydrodynamic interaction should be taken into account, e.g., Equations (7) and (10). The hydrodynamic data acquired at different concentrations then provide suitable orthogonal information, e.g., by comparing Equations (1) and (9). For a more detailed and recent assessment of the described phenomenology in combination with other hydrodynamic and light scattering techniques the reader may refer to reference [12].

## 3. The Hydrodynamic Invariant and Sedimentation Parameter

**Scheme 1:**
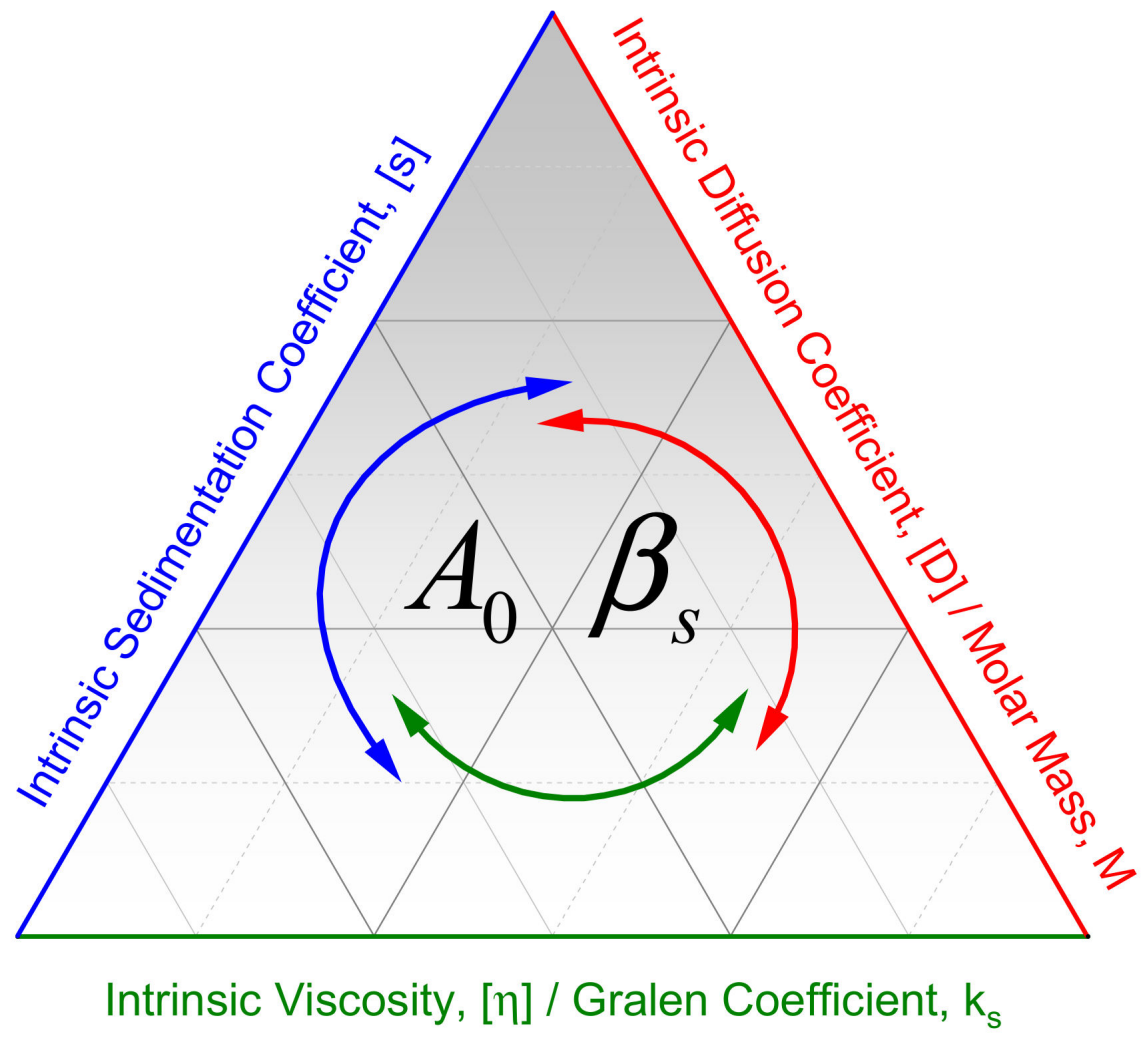
Graphical representation of the interplay of the basic components of the hydrodynamic invariant, $A_{0}$, and sedimentation parameter, $\;\beta_{s}$, with the experimental intrinsic viscosity, $\left[\eta \right]$ (Equation (4)), its translational friction analog the Gralen coefficient, $k_{s}$ (Equation (7)), the intrinsic sedimentation coefficient, $\left[s\right]$ (Equation (8)), and the intrinsic diffusion coefficient, $\left[D\right]$ (Equation (11)), respectively the molar mass of the macromolecules, M.

After having discussed the individual hydrodynamic characteristics and the governing experimental requirements and calculations for their establishment (vide supra), we now move toward the very basic definition of the hydrodynamic invariant and sedimentation parameter. This is followed by an overview of respective values selected from the recent literature, primarily over the last decade. Thereby, we mainly consider aqueous macromolecule systems with potential for pharmaceutical applications. To alleviate the discussion, Scheme 1 depicts all the (intrinsic) estimates on each of the sides of a triangle.

Historically, the hydrodynamic invariant may be traced back to initial studies from Mandelkern and Flory published in 1952, who noted that the product $\left[s\right]{\left[\eta \right]}^{1/3}M^{-2/3}$ should be the same for all randomly coiled macromolecules [15]. The authors utilized literature data and found agreement within the experimental error for polystyrene and cellulose acetate fractions. Interestingly, in 1953, a study initially presented in 1952 by Tsvetkov and Klenin appeared [16]. The authors found invariance in fractionated samples of polystyrene in solvent dichloroethane by calculating the product $\eta_{0}D{\left(M\left[\eta \right]\right)}^{1/3}$ for the various fractions. These initial notions are remarkably similar to what we nowadays refer to as the hydrodynamic invariant. The perception of such a hydrodynamic invariant can be found in a comprehensive review of experimental hydrodynamic data that was published by Tsvetkov et al. in his seminal contribution in 1984 [17]. The basic components of the hydrodynamic invariant $A_{0}$ (Scheme 1) can be interrelated as follows:

(12) $$A_{0}={\left(R\left[s\right]{\left[D\right]}^{2}\left[\eta \right]\right)}^{1/3}={\left(M\left[\eta \right]\right)}^{1/3}\left[D\right]=R\left[s\right]{\left[\eta \right]}^{1/3}M^{-2/3}$$

where the experimentally determined terms are defined by Equations (4), (8), and (11), and R is the universal gas constant. We note that Equation (12) traditionally utilizes values of the intrinsic viscosities, $\left[\eta \right]$ (Equation (4)) in numerical values ${\mathrm{dLg}}^{-1}$, i.e., estimates in ${\mathrm{cm}}^{3}g^{-1}$ are divided by a factor of 100.

The hydrodynamic invariant, $A_{0}$ (Equation (12)), can therefore be determined by a suitable selection of data available for its calculation and typically requires a set of sophisticated and orthogonal experimental techniques on the same sample for its establishment, i.e., a single experimental technique is typically insufficient. This is because of its orthogonal experimental nature. As the name implies, a homologous series of macromolecules, or macromolecules of similar interplaying solution properties, should lead to similar values of the hydrodynamic invariant, $A_{0}$ (Equation (12)), fluctuating around their mean since, e.g., a change in the intrinsic sedimentation coefficients, $\left[s\right]$ (Equation (8)), of macromolecules necessarily results in a respective change of at least one of the other components of the invariant, primarily two [17]. An exception to this may be found with hyperbranched macromolecular structures that were shown to only moderately providing a change in intrinsic viscosities, $\left[\eta \right]$, when one, respectively two, of the other properties is modulated [24]. 

The major components of the hydrodynamic invariant, $A_{0}$ (Equation (12)), can be classified in translational and rotational components, i.e., by the intrinsic viscosity, $\left[\eta \right]$ (Equation (4)), the intrinsic sedimentation coefficient, $\left[s\right]$ (Equation (8)), and the intrinsic diffusion coefficient, $\left[D\right]$ (Equation (11)), the first being of rotational nature, the latter two being of translational nature. We note that in the framework of hydrodynamics, the intrinsic diffusion coefficient, $\left[D\right]$, can also be substituted by the molar mass of the macromolecules, M. Through the classical Svedberg equation, the molar mass of macromolecules can be calculated by knowledge of the intrinsic sedimentation coefficient, $\left[s\right]$ (Equation (8)), and intrinsic diffusion coefficient, $\left[D\right]$ (Equation (11)):

(13) $$M=M_{s,D}=R\frac{\left[s\right]}{\left[D\right]}.$$

The substitution of $\left[s\right]$ or $\left[D\right]$ from this equation into Equation (12) enables the mathematical transformation of the individual expressions of the hydrodynamic invariant, $A_{0}$ (Equation (12)). 

Furthermore, there are upper and lower theoretical and experimental values of the hydrodynamic invariant, $A_{0}$ (Equation (12)), which can be assumed. These are useful for the verification of the adequacy of experimental hydrodynamic data. The lower limit from a solid impermeable sphere is straightforward by theory and assumes a value of $A_{0}=2.9\;\times \;10^{-10}\;{\mathrm{gcm}}^{2}s^{-2}K^{-1}{\mathrm{mol}}^{-1/3}$, based on the very basic definition of the $\left[\eta \right]$, $\left[s\right]$, and $\left[D\right]$ values of spherical particles [17].

In its perhaps more abstract theoretical form, the hydrodynamic invariant, $A_{0}$ (Equation (12)), may be calculated by the Boltzmann constant, $k_{B}$, and the Flory hydrodynamic parameters ϕ and P, i.e., $A_{0}=k_{B}{\left(\phi_{0}/100\right)}^{1/3}P_{0}^{-1}$, where the index zero represents a limiting value of the respective parameters. Interestingly, by utilizing different values of the Flory hydrodynamic parameter, $\phi_{0}$ (in units ${\mathrm{mol}}^{-1}$), obtained by different theories and by assuming different values of the Flory hydrodynamic parameter, $P_{0}$ (dimensionless), one arrives at different theoretical values for $A_{0}$. The upper is represented by $A_{0}=4.1\;\times \;10^{-10}\;{\mathrm{gcm}}^{2}s^{-2}K^{-1}{\mathrm{mol}}^{-1/3}$ [17]. Typical experimental values for macromolecules are discussed later in line with the theoretical lower limit of a solid impermeable sphere (vide supra) and the upper limiting value. Noted disagreement between purely theoretical and statistically-averaged experimental values are also discussed.

The very concept of the sedimentation parameter, $\beta_{s}$, may be associated with experimental efforts and theoretical considerations of Pavlov et al. [18,19]. It can be seen as a re-incarnation of the hydrodynamic invariant, $A_{0}$ (Equation (12)), i.e., a conceptual extension or, better, analogue (Scheme 1). Here, the translational friction analogue to the intrinsic viscosity, $\left[\eta \right]$ (Equation (4)), the Gralen coefficient $k_{s}$ (Equation (7)), having the same physical unit as $\left[\eta \right]$, is utilized. In this case, $\beta_{s}$ only considers translational friction phenomena by utility of the concentration dependence of the sedimentation coefficient, s (Equation (7)). We have the following definitions for $\beta_{s}$:

(14) $$\beta_{s}=k_{B}^{-2/3}{\left(N_{A}\left[s\right]{\left[D\right]}^{2}k_{s}\right)}^{1/3}={\left(Mk_{s}\right)}^{1/3}\left[D\right]k_{B}^{-1}=N_{A}\left[s\right]k_{s}^{1/3}M^{-2/3}\;.$$

As for $A_{0}$ (Equation (12)), the intrinsic diffusion coefficient, $\left[D\right]$, can straightforwardly be replaced by the molar mass of the macromolecule, M, since the universal gas constant, R, in Equation (13) is defined by the product of the Boltzmann constant, $k_{B}$, and the Avogadro constant, $N_{A}$, i.e., $R=k_{B}N_{A}$. Substitution of $\left[s\right]$ (Equation (8)) or $\left[D\right]$ (Equation (11)) from Equation (13) in the form, $M=M_{s,D}=\;k_{B}N_{A}\left[s\right]/\left[D\right]$, allows mathematical transformation of the individual expressions of the sedimentation parameter, $\beta_{s}$ (Equation (14)).

Once values of the hydrodynamic invariant, $A_{0}$ (Equation (12)), are established, the corresponding parameters for calculation of the sedimentation parameter, $\beta_{s}$ (Equation (14)), are typically available. In cases, the sedimentation coefficient, s (Equation (7)), practically shows no concentration dependence [13,14]. Furthermore, in examples of only a weak concentration dependence, as is the case for a rather limited and low molar mass range, [13,14] or virtually ideally behaving systems, the error for the estimation of $k_{s}$ (Equation (7)) is expected to be very large. Such systems, therefore, escape a proper analysis in the framework of the sedimentation parameter, $\beta_{s}$ (Equation (14)).

Having the above-made definitions for $A_{0}$ (Equation (12)) and $\beta_{s}$ (Equation (14)) at hand, we can move to their practical utility by discussing recently published values or values calculated from the published data. To the majority, the publications cover the period from the first comprehensive publication of Tsvetkov et al. [17] to the present.

### 3.1. Recent Values for the Hydrodynamic Invariant, $A_{0}$

**Figure 1:**
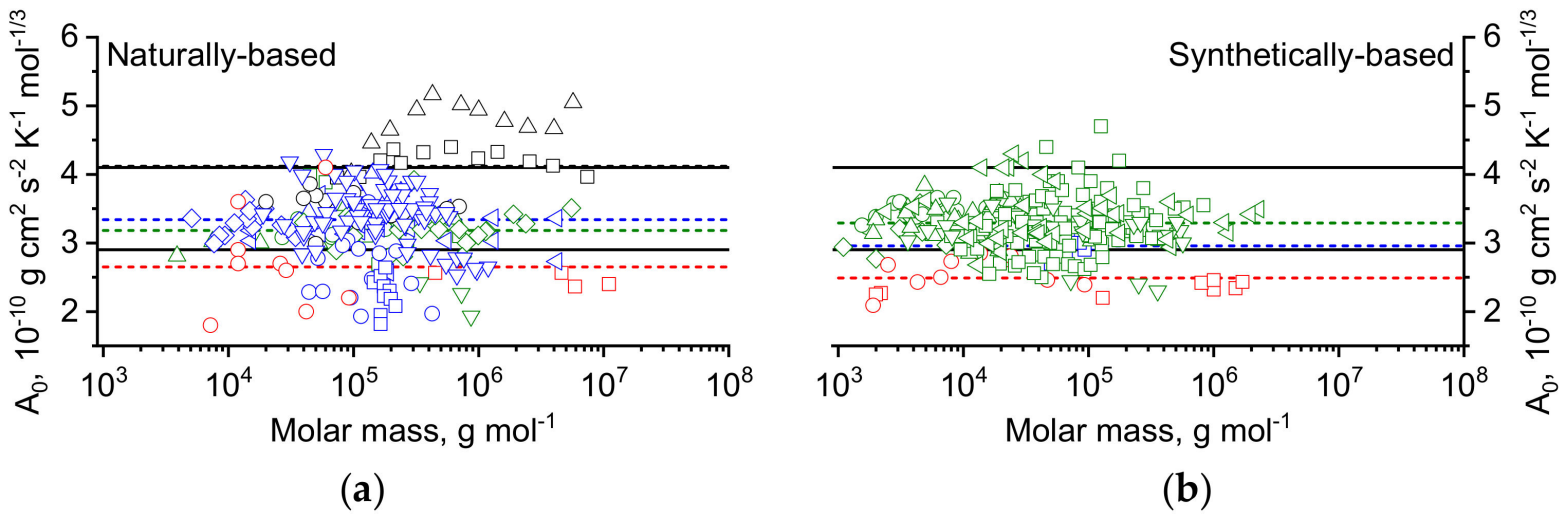
Semi-logarithmic plot of the hydrodynamic invariants, $A_{0}$ (Equation (12)), against molar mass from the literature during the last decade, divided in (**a**) naturally-based and (**b**) synthetically-based macromolecules. Shown also are the lower limiting value of the solid impermeable sphere and the theoretical upper limit by the solid black lines. The values for the hydrodynamic invariants, $A_{0}$ (Equation (12)), were either published directly by the authors or calculated by us using the data provided. Dashed lines represent average values for the hydrodynamic invariants, $A_{0}$ (Equation (12)), calculated from the respective data of a particular conformational type of macromolecules. Color and symbol assignments for naturally-based macromolecules: Black—Rigid, Xanthan (squares) [25], Chitosan (circles) [26,27], Schizophyllan (triangles up) [28]; Blue—Semi-Flexible, Pectin (squares) [29,30], Chitosan (circles) [31,32,33,34], Chitin (triangles up) [35], Cellulose (triangles down) [12,36,37,38,39,40,41], Heparin (diamonds) [42], Ficoll400 (triangles left) [43]; Green—Flexible, Glucan (squares) [44], Mannan (circles) [45], Pullulan (triangles up) [46], Carrageenan (triangles down) [47], Polyvinylsaccharide (diamonds) [48,49]; Red—Branched, Glucan (squares) [50], Lysin (circles) [51]. Color and symbol assignments for synthetically-based macromolecules: Blue—Semi-Flexible, Polyelectrolyte (squares), cationic [52]; Green—Flexible, Polyelectrolyte (squares), cationic [53,54,55,56,57,58,59,60,61,62], anionic [63], Poly(ethylene) glycol (PEG) (circles) [14], Poly(2-alkyl-2-oxazoline) (POx)/Poly(oligo(2-ethyl-2-oxazoline)) based polymers (triangles up) [13,64,65], amide containing vinyl copolymers (triangles down) [66,67], Poly(ethylene) imine (PEI) (diamonds) [68], Polyvinylpyrrolidone (triangles left) [69,70,71,72]; Red—Branched, PEG copolymers (squares) [24], Dendrimers (circles) [73,74].

Figure 1 shows the hydrodynamic invariants, $A_{0}$ (Equation (12)), of (a) naturally-based macromolecules and (b) synthetically-based, i.e., artificial, macromolecules. The hydrodynamic invariants, $A_{0}$ (Equation (12)), were published by the authors themselves or calculated by us making use of the published data. On first sight, all data show random fluctuations of $A_{0}$ (Equation (12)) against molar mass in an apparently non-systematic data cloud, in a range of approximately $A_{0}\approx \left(2-5\right)\;\times \;10^{-10}\;{\mathrm{gcm}}^{2}s^{-2}K^{-1}{\mathrm{mol}}^{-1/3}$.

Shown also in Figure 1 are the lower limiting values of the solid impermeable sphere and the upper theoretical limit by solid black lines. Globally, the majority of the data falls within the given upper and lower limits and an apparent influence of the molar mass on the data appears absent. Apparent also is that data points are interspersed between different evident conformations of the macromolecules. For a better representation of statistics, we calculated average values of $A_{0}$ (Equation (12)) of the different subclasses shown by the dashed lines. Table 1 comprehends the average values, while distinction is made between macromolecular system and origin types.

It is apparent, that macromolecules with a flexible backbone assume average values of $A_{0}=\left(3.2-3.3\right)\;\times 10^{-10}\;{\mathrm{gcm}}^{2}s^{-2}K^{-1}{\mathrm{mol}}^{-1/3}$ (considering hydrodynamic invariants in an overall broad range of $A_{0}\approx \left(2-5\right)\;\times \;10^{-10}\;{\mathrm{gcm}}^{2}s^{-2}K^{-1}{\mathrm{mol}}^{-1/3}$, Equation (12)), i.e., also lying outside the theoretical limits. Both naturally-based and synthetically-based macromolecules appear readily similar. 

The (hyper-)branched macromolecules show average values in a range of $A_{0}=\left(2.5-2.7\right)\;\times 10^{-10}{\;\mathrm{gcm}}^{2}s^{-2}K^{-1}{\mathrm{mol}}^{-1/3}$, again in the same range for naturally- and synthetically-based macromolecules. By means of statistics, the $A_{0}$ values are systematically smaller than that of solid, impermeable spheres ($A_{0}=2.9\;\times \;10^{-10}{\;\mathrm{gcm}}^{2}s^{-2}K^{-1}{\mathrm{mol}}^{-1/3}$), an aspect that has long puzzled researchers [24,73,74,75]. It appears to be associated with the spherical-like/globular conformations being quasi permeable to the solvent.

The distinction between flexible and semi-flexible macromolecules may also be made, but we doubt whether it makes sense to provide explicit values, as the range is rather interspersed and difficult to classify. As might be expected, the $A_{0}$ values (Equation (12)) for semi-flexible conformations is statistically slightly larger than the value for flexible backbone macromolecules in naturally-based macromolecules (blue dashed line in Figure 1a). Again, we see a smaller $A_{0}$ value (Equation (12)) for semi-flexible conformations in synthetically-based macromolecules (blue dashed line in Figure 1b). However, this average value is only based on one publication, which is why the global statistics cannot be applied. 

Generally noting the relatively large variation in the data, these still underline the global power of the hydrodynamic invariant approach, $A_{0}$ (Equation (12)), in the verification of the interplay of prime hydrodynamic parameters established by Equations (4), (8), and (11) (Scheme 1). Furthermore, the noted agreement between the origin of macromolecules (naturally- or synthetically-based) re-affirms that the very basic concepts of the physical solution structure (Scheme 1) do not depend on origin, chemical composition, and to some extend on the quality of the solvent for the macromolecules. In perception, once hydrodynamic invariants, $A_{0}$ (Equation (12)), above or below the theoretical range of invariants, $A_{0}=\left(2.9-4.1\right)\;\times \;10^{-10}\;{\mathrm{gcm}}^{2}s^{-2}K^{-1}{\mathrm{mol}}^{-1/3}$ are calculated, the accuracy of the primarily determined hydrodynamic characteristics by Equations (4), (8), and (11), respectively, and the values of the molar mass estimated by any technique of hydrodynamics and light scattering, should be checked for accuracy. A perhaps valid exception to this rule of thumb is represented by (hyper-)branched architectures (Table 1).

### 3.2. Recent Values for the Sedimentation Parameter, $\beta_{s}$

**Figure 2:**
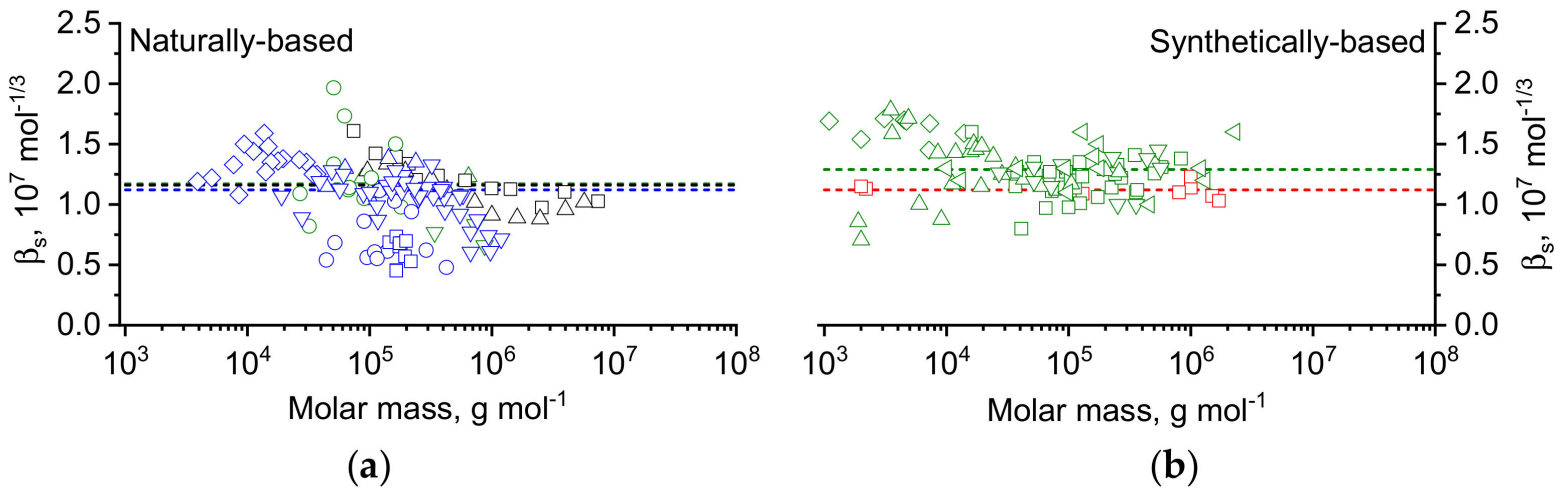
Semi-logarithmic plot of the sedimentation parameter, $\beta_{s}$ (Equation (14)), from different publications during the last decade, divided in (**a**) naturally-based and (**b**) synthetically-based macromolecules. The values for the sedimentation parameters were either published directly by the authors or calculated by us using the data provided. Dashed lines represent different averages for the sedimentation parameter, $\beta_{s}$ (Equation (14)), characteristic for different conformation types. Color and symbol assignments of naturally-based macromolecules: Black—Rigid, Xanthan (squares) [25], Schizophyllan (triangles up) [28]; Blue—Semi-Flexible, Pectin (squares) [29,30], Chitosan (circles) [31,33,34], Chitin (triangles up) [35], Cellulose (triangles down) [12,38,39,40,41,77], Heparin (diamonds) [42]; Green—Flexible, Mannan (circles) [45,78], Pullulan (triangles up) [46], Carrageenan (triangles down) [47]. Color and symbol assignments of synthetically-based macromolecules: Green—Flexible, Polyelectrolyte (squares), cationic [55,59,60], POx (triangles up) [13,65], amide containing vinyl copolymers (triangles down) [66,67], PEI (diamonds) [68], Polyvinylpyrrolidone (triangles left) [69]; Red—Branched, PEG copolymers (squares) [24].

On the basis of the analogy put forward, between the hydrodynamic invariant, $A_{0}$ (Equation (12)), and sedimentation parameter, $\beta_{s}$ (Equation (14)), we as well comprehend values of the latter and compare them against each other. Typically, if proper estimations for the invariant are possible, then also values for the sedimentation parameter can be established, except no concentration dependence of the sedimentation coefficient, s (Equation (7)), or a very minor dependence is observed. Figure 2 shows the sedimentation parameters, $\beta_{s}$, of (a) naturally-based and (b) synthetically-based macromolecules. As for the hydrodynamic invariant, $A_{0}$ (Equation (12)), the sedimentation parameters, $\beta_{s}$ (Equation (14)), were published by the authors themselves or calculated by us using the published data according to Equation (14). Primarily, the publications cover the period from the publications of Pavlov et al. in 1988 and 1995 [18,19] to the present.

The sedimentation parameter, $\beta_{s}$ (Equation (14)), is rather less frequently published compared to the hydrodynamic invariant, $A_{0}$ (Equation (12)). For the calculation of the sedimentation parameter, the Gralen coefficient, $k_{s}$ (Equation (7)), is utilized. This also implies the use of several concentrations, over suitable concentration ranges, used for the extrapolation of the sedimentation coefficient, s, to infinite dilution (Equation (7)). Here, such experiments of sedimentation velocity could establish a relation to the conformation. On a practical note, outside the scope of the present review, an interrelation between rotational (Equation (1)) and translational friction (Equation (9)) is referred to as the Wales-van-Holde ratio, [76] also assuming characteristic values for the macromolecules of certain conformation.

As observed in plots of the hydrodynamic invariants, $A_{0}$ (Equation (12)), the values for the sedimentation parameters, $\beta_{s}$ (Equation (14)), fluctuate significantly more for the naturally-based macromolecules than for the synthetically-based macromolecules (Figure 2). Again, this is an issue that depends on the appropriate degree of dilution used for their establishment and existent non-idealities, due to dispersity and structural considerations [12]. Additionally, the data do not appear to allow conclusions on the molar mass dependence of the sedimentation parameter.

In 1988 and 1995 Pavlov et al. [18,19] reported values for the sedimentation parameters dependent on the conformation. For flexible macromolecules, a value of $\beta_{s}=1.2\;\times \;10^{7}\;{\mathrm{mol}}^{-1/3}$ and for rigid and spherical conformations, a value of $\beta_{s}=1.0\;\times \;10^{7}\;{\mathrm{mol}}^{-1/3}$ was published. Due to the limited literature sources in recent years, we can only provide average values for flexible and (hyper-)branched conformations of synthetically-based macromolecules. For the flexible conformations, we calculate an average value of $\beta_{s}=1.29\;\times \;10^{7}\;{\mathrm{mol}}^{-1/3}$ and a value of $\beta_{s}=1.12\;\times \;10^{7}\;{\mathrm{mol}}^{-1/3}$ for (hyper-)branched macromolecule conformations. For the naturally-based macromolecules, we calculate mean values for a rigid and flexible conformation, where rigid macromolecules ($\beta_{s}=1.16\;\times \;10^{7}\;{\mathrm{mol}}^{-1/3}$) provide a similar value than flexible macromolecule conformations with $\beta_{s}=1.17\;\times \;10^{7}\;{\mathrm{mol}}^{-1/3}$. The values for flexible conformations are typical in an overall broad range of approximately $\beta_{s}\approx \left(0.5-2.0\right)\;\times \;10^{7}\;{\mathrm{mol}}^{-1/3}$, showing no obvious dependence on the origin and molar mass of the macromolecules. 

As mentioned for the hydrodynamic invariant, $A_{0}$ (Equation (12)), average errors are large and calculated values are primarily useful by accepting their statistical nature. It is, therefore, rather advisable to treat values of the sedimentation parameter, $\beta_{s}$ (Equation (14)) with even more care than the hydrodynamic invariant, $A_{0}$ (Equation (12)). Notwithstanding, and as noted above, physically-sound values of the sedimentation parameter, $\beta_{s}$ (Equation (14)), provide indication for the adequacy of individually-determined characteristics of the macromolecules of study, or necessity for re-iteration of accuracy of individual characteristics determined by the different techniques, including molar mass estimations by the methods of hydrodynamics or light scattering. 

Table 2 displays average values of the sedimentation parameter, $\beta_{s}$ (Equation (14)) from the present work, separated according to knowledge of the physical structure of the respective macromolecular system.

## 4. Global Discussion

The prime important and very practical utility of the hydrodynamic invariant, $A_{0}$ (Equation (12)), and sedimentation parameter, $\beta_{s}$ (Equation (14)), stems from the verification of hydrodynamic data according to the fundamental concepts of experimental macromolecule physics (Scheme 1). Table 3 shows data of the hydrodynamic invariant, $A_{0}$ (Equation (12)), comprehended by Tsvetkov et al. in a study published in 1984 [17]. As well contained in Table 3 are typical values for the sedimentation parameter, $\beta_{s}$ (Equation (14)) according to Pavlov et al. [18,19]. The second column shows average values of $A_{0}$ (Equation (12)) and the fourth column average values of $\beta_{s}$ (Equation (14)) from the data presented in this short review. No distinction is made between the naturally- and synthetically-based macromolecules; above we noted their statistical similarity.

Surprisingly, the data for the hydrodynamic invariant, $A_{0}$ (Equation (12)) appear pretty close, more than 30 years after the initially performed statistical averaging [17]. The values of the sedimentation parameter, $\beta_{s}$ (Equation (14)), without distinction between the naturally- and synthetically-based macromolecules, are as well in a similar range as those published by Pavlov et al. [18,19]. 

With the above-presented analysis, the here outlined concept appears validated compared to previous establishments. This provides the most fundamental incentive of using such a concept in establishing the molecular hydrodynamic behavior of new macromolecular solution structures accessible by modern synthetic methods, or re-affirming the properties of known macromolecular solution structures that are tested for the adequacy of molar mass estimations [12,13,14].

Notwithstanding, and as for every concept, there are limitations. In the event of the accumulation of many data sources and statistical averages, data become more confined to statistically true values. However, for a particular study, and a limited amount of different molar masses to establish the hydrodynamic invariant, $A_{0}$ (Equation (12)), or the sedimentation parameter, $\beta_{s}$ (Equation (14)), significant deviations are likely to occur. These shall briefly be discussed. 

In the statistical description of macromolecular chains put forward in very initial rationales, the mean square end-to-end distance, $\langle h^{2}\rangle$, represents a result of averaging over all chain conformations (Equations (1)–(3)). Strictly speaking, this is an ideal situation [1]. This means it is a statistical property for a discrete species of a singular molar mass value by itself. Except perhaps natural proteins/enzymes or DNA/RNA, macromolecules are typically disperse in nature, and show more pronounced solution non-idealities compared to well-defined species such as proteins, i.e., they show variability in their number of repeating units and/or chemical composition and/or backbone charges next to other structural non-idealities [12]. These variations all affect statistical average properties of such populations in solution. Practically, this also means that individual estimates by Equations (4), (8), and (11) themselves are statistical in nature, because they originate from typically observed distributions of properties assigned to a single estimate, e.g., by calculating suitable distribution moments. Errors or bias in estimating each of the hydrodynamic characteristics for highly disperse or even heterogeneous macromolecule populations are, therefore, very likely to impact the later established accuracy of the value of the hydrodynamic invariant, $A_{0}$ (Equation (12)), or sedimentation parameter, $\beta_{s}$ (Equation (14)). Such errors in individual estimates may potentially amplify in nature or statistically average out [12]. The largest error is expected from inaccurately determined intrinsic diffusion coefficients, $\left[D\right]$ (Equation (11)), that are notoriously difficult to determine in an absolute manner, particularly for disperse samples and the required degrees of dilution [12]. In any of the put forward estimations, the intrinsic diffusion coefficient, $\left[D\right]$ (Equation (11)), typically reveals the highest exponential dependence in the expressions for the $A_{0}$ (Equation (12)) and $\beta_{s}$ values (Equation (14)). 

Notwithstanding that such errors cannot be circumvented, the different techniques to establish the hydrodynamic invariant, $A_{0}$ (Equation (12)), and sedimentation parameter, $\beta_{s}$ (Equation (14)), help to verify the interplay between them (Scheme 1). Using a sophisticated set of highly orthogonal experimental techniques, this may even be possible for highly disperse macromolecule populations [12]. As a consequence, it appears advisable, that once the parametric estimates of particular macromolecule populations are available, such a concept shall be applied and the results reported. This can help in the identification of possible errors in estimating individual parameters contributing to the establishment of the hydrodynamic invariant, $A_{0}$ (Equation (12)), or sedimentation parameter, $\beta_{s}$ (Equation (14)). Most importantly, this can also help with verifying the adequacy of molar mass estimations in concert with several hydrodynamic characteristics based on rotational and translational friction of macromolecules [12,13,14]. Again, this is a statistical issue of identification by a suitable set of orthogonal experiments. The more orthogonal experimental efforts that are combined, the better the likelihood of accuracy of the determined correctness of derived characteristics, may it be simply the absolute molar mass. Unsurprisingly, very well-defined macromolecule populations known from modern precision synthesis provide the highest accuracy of hydrodynamic characteristics, molar mass estimates, and averages of the hydrodynamic invariant, $A_{0}$ (Equation (12)), or sedimentation parameter, $\beta_{s}$ (Equation (14)) [13,14,65].

Despite the many carefully considered advantages, the following obstacles should be made aware to the experimentalist. General concerns may be comprehended by:

- Dispersity/heterogeneity in macromolecule populations. This aspect is of critical importance since each of the techniques applied for macromolecular characterization may show some bias toward dispersity and existence of subpopulations, i.e., heterogeneity. A classic example of such is the utilization of light scattering on very disperse populations, or disperse small molar mass samples, that may tend to bias average molar mass estimations, particularly if no prefractionation/separation is applied [12,13,79,80]. Care, should therefore, be taken to identify potentially all components of a population by high performance separation and preferably concentration-sensitive detection [13].
- Correct choice of experimental conditions to investigate non-ideality phenomena. A classical and contemporary example is associated to the degree of dilution in hydrodynamic measurements, possible to be defined by the Debye Parameter as the product of intrinsic viscosity, $\left[\eta \right]$, in units ${\mathrm{cm}}^{3}g^{-1}$ and mass concentration, c, in units ${\mathrm{gcm}}^{-3}$ [12]. Associated effects observed in sedimentation velocity experiments are potentially occurring sedimentation boundary sharpening phenomena [81] and/or the Johnston–Ogston effect [82]. To identify such cases, the measurement of intrinsic viscosities, $\left[\eta \right]$ (Equation (4)) appears necessary, and anomalies in sedimentation profiles be identified by checking sedimentation boundary behavior at different degrees of dilution [12].
- Polyelectrolytes are inherently difficult to study. The sufficient compensation of macromolecular backbone charge effects, necessary to reduce difficulties in the measurements by sedimentation velocity experiments and the typically observed non-linearity of the Huggins- and Kraemer extrapolation procedures for estimations of the intrinsic viscosity, $\left[\eta \right]$, are critical [54,55,68]. At present, the detailed hydrodynamic study and interrelation of hydrodynamic characteristics within the framework of the hydrodynamic invariant, $A_{0}$ (Equation (12)), or sedimentation parameter, $\beta_{s}$ (Equation (14)), are still awaiting validation and establishment. Under conditions of sufficient screening of charged moieties in macromolecules, the behavior of polyelectrolytes should follow that of their non-charged analogues. In the presence of varying electrostatic interactions, the conformational properties can vary [12].

It, therefore, may not appear surprising that the concept of the hydrodynamic invariant, $A_{0}$ (Equation (12)) or sedimentation parameter, $\beta_{s}$ (Equation (14)), represent relatively high error margins dependent on the non-ideality, dispersity, and heterogeneity of the macromolecular samples. Despite this very fact, such interrelation appears robust throughout the reported literature of naturally- and synthetically-based macromolecules, once statistics on large amounts of sample are applied. This concept can then also help to verify the determination of absolute molar mass values established for any desirable macromolecular structure, finding its origin in the interplay of macromolecular hydrodynamic parameters, at least by a qualitative test of accuracy (Equations (12) and (14), Scheme 1). 

An interesting statistical approach to the actual molar mass of synthetic macromolecules can, therefore, be developed on the basis of the hydrodynamic invariant $A_{0}$ (Equation (12)) or sedimentation parameter, $\beta_{s}$ (Equation (14)), given that a set of primary hydrodynamic characteristics are determined. This is accessible by re-arrangement of Equations (12) and (14), and by utilizing average estimates of $A_{0}$ and $\beta_{s}$ values reported in Table 3 [12]. The advantage of such a statistical estimate of molar masses clearly emanates from utilizing additional hydrodynamic descriptors for their calculation, i.e., being more robust for the representation of the different rotational and translational friction properties of the macromolecules (Scheme 1). In using such an attempt, one should be aware of the statistical nature of molar masses [12].

Finally, we note that the classically established empirical scaling relationships of the primary hydrodynamic characteristics ($\left[\eta \right]$, s, and D) to the molar mass and their interrelation can be expected valid, once values of the hydrodynamic invariant, $A_{0}$ (Equation (12)), or the sedimentation parameter, $\beta_{s}$ (Equation (14)), are successfully established [12,13,14].

## 5. Conclusions

In this short review, we discussed and evaluated recent results reported in publications concerning solution hydrodynamics of synthetically- and naturally-based macromolecules. Along that line, we attempted the contextualization of the results with the concept of the hydrodynamic invariant and sedimentation parameter. 

We found that average values for the hydrodynamic invariant and sedimentation parameter, when compared to the first cumulative literature data, are in fundamental agreement. This situation is independent of the chemical nature and origin of the macromolecule populations and of the molar mass to be verified. This further supports the necessity and usefulness of interrelating all primary hydrodynamic parameters of macromolecules and colloidal objects such as intrinsic viscosity, intrinsic sedimentation coefficient, and intrinsic diffusion coefficient, in order to pin down any substantiated conclusion about a particular macromolecular sample population at hand, may it be naturally- or synthetically-based macromolecules.

Last but not least, the hydrodynamic invariant and the sedimentation parameter are the one and only opportunity for the validation of accurate molar mass estimations of naturally- or synthetically originating macromolecules by the methods of macromolecular hydrodynamics and light scattering, particularly if differences of molar mass estimations are observed between the different techniques.

## Figures and Tables

**Table 1:** Average values of the hydrodynamic invariant, $A_{0}$ (Equation (12)) in $10^{-10}{\mathrm{gcm}}^{2}s^{-2}K^{-1}{\mathrm{mol}}^{-1/3}$, from the data comprehended in the present review.

Macromolecular System	Naturally-Based	Synthetically-Based
Flexible	3.18±0.35	3.29± 0.37
Rigid	4.12±0.56	−
(Hyper-)branched	2.65±0.59	2.49±0.24

**Table 2:** Average values of the sedimentation parameter, $\beta_{s}$ (Equation (14)), in $10^{7}{\mathrm{mol}}^{-1/3}$ from the data comprehended in the present review.

Macromolecular System	Naturally-Based	Synthetically-Based
Flexible	1.17±0.32	1.29±0.22
Rigid	1.16±0.18	−
(Hyper-)branched	−	1.12±0.06

**Table 3:** Average values of the hydrodynamic invariant, $A_{0}$ (Equation (12)) in $10^{-10}{\mathrm{gcm}}^{2}s^{-2}K^{-1}{\mathrm{mol}}^{-1/3}$, and sedimentation parameter, $\beta_{s}$ (Equation (14)) in $10^{7}{\mathrm{mol}}^{-1/3}$, from the literature and averages from the data comprehended in the present review.

Macromolecular System	$a^{\;}\;A_{0}$	$A_{0}$	$b^{\;}\;\beta_{s}$	$\beta_{s}$
Flexible	3.2±0.2	3.3±0.4	1.25	1.27±0.24
Rigid	3.7±0.4	4.1±0.6	1.00	1.16±0.18
(Hyper-)branched	-	2.7±0.4	-	1.12±0.06
Sphere	2.9	-	-	-

^a^ Average values taken from Tsvetkov et al. [17]. ^b^ Average values taken from Pavlov et al. [18,19].
